# Diesel exhaust modulates ozone-induced lung function decrements in healthy human volunteers

**DOI:** 10.1186/s12989-014-0037-5

**Published:** 2014-09-02

**Authors:** Michael C Madden, Tina Stevens, Martin Case, Michael Schmitt, David Diaz-Sanchez, Maryann Bassett, Tracey S Montilla, Jon Berntsen, Robert B Devlin

**Affiliations:** 1 EPHD, NHEERL, US EPA, Research Triangle Park, Chapel Hill 27711, NC, USA; 2 TRC Environmental, Chapel Hill 27514, NC, USA; 3 Currently ORISE, Research Triangle Park, Chapel Hill 27711, NC, USA; 4 U.S EPA Human Studies Facility, 104 Mason Farm Road, Chapel Hill 27599-7315, NC, USA

**Keywords:** Diesel exhaust, Ozone, Co-exposure, Lung function, Greater than additive effects

## Abstract

The potential effects of combinations of dilute whole diesel exhaust (DE) and ozone (O_3_), each a common component of ambient airborne pollutant mixtures, on lung function were examined. Healthy young human volunteers were exposed for 2 hr to pollutants while exercising (~50 L/min) intermittently on two consecutive days. Day 1 exposures were either to filtered air, DE (300 μg/m^3^), O_3_ (0.300 ppm), or the combination of both pollutants. On Day 2 all exposures were to O_3_ (0.300 ppm), and Day 3 served as a followup observation day. Lung function was assessed by spirometry just prior to, immediately after, and up to 4 hr post-exposure on each exposure day. Functional pulmonary responses to the pollutants were also characterized based on stratification by glutathione S-transferase mu 1 (GSTM1) genotype. On Day 1, exposure to air or DE did not change FEV1 or FVC in the subject population (n = 15). The co-exposure to O_3_ and DE decreased FEV1 (17.6%) to a greater extent than O_3_ alone (9.9%). To test for synergistic exposure effects, i.e., in a greater than additive fashion, FEV1 changes post individual O_3_ and DE exposures were summed together and compared to the combined DE and O_3_ exposure; the p value was 0.057. On Day 2, subjects who received DE exposure on Day 1 had a larger FEV1 decrement (14.7%) immediately after the O_3_ exposure than the individuals’ matched response following a Day 1 air exposure (10.9%). GSTM1 genotype did not affect the magnitude of lung function changes in a significant fashion. These data suggest that altered respiratory responses to the combination of O_3_ and DE exposure can be observed showing a greater than additive manner. In addition, O_3_-induced lung function decrements are greater with a prior exposure to DE compared to a prior exposure to filtered air. Based on the joint occurrence of these pollutants in the ambient environment, the potential exists for interactions in more than an additive fashion affecting lung physiological processes.

## 1 Introduction

Numerous epidemiological studies have demonstrated an association between short-term exposure to ambient airborne particulate matter (PM) and adverse cardiopulmonary effects including premature mortality, increased hospitalizations for lung problems including infections, exacerbation of asthma symptoms, chronic bronchitis, and hospitalization for clinical cardiac events including arrhythmias, myocardial infarctions, and congestive heart failure [1],[2]. The health effects are more strongly associated with PM that is smaller than 2.5 μm, i.e. PM_2.5_, which typically is derived from human based activities such as vehicular emissions. PM_2.5_ is a complex mixture of organic and inorganic compounds absorbed onto carbonaceous material with the composition varying across space and time. In this complex mixture of ambient air substances, the ubiquitous pollutants ozone (O_3_) and diesel exhaust (DE) can be can be major and important components. DE can have “hotspots” such as bus terminals and major streets [3]. Levels of DE PM_2.5_ reached transient concentrations of several hundred μg/m3 during drive-by studies [4]. O_3_ levels have generally been decreasing in the US, but can reach over 0.1 ppm on a regular basis.

Ozone is one of the best studied gas phase pollutants in terms of lung biological effects. With dozens of controlled human exposures, biological responses have been well documented particularly for induction of decrements of lung function and inflammation typically expressed as decreased FEV1 and FVC [5] and lung neutrophilia [6],[7]. in a concentration-response manner (typically 0.08-0.4 ppm). Repeated exposure studies with healthy young adults inhaling 0.4 ppm O_3_ for 2 hrs have shown a decrement in lung function after the first exposure and an even greater decrement after a second day exposure, but then attenuated lung function decrements for the next 3 exposure days [8],[9]. Some reports have linked certain genetic polymorphisms to possible sensitivities to O_3_ exposure. For example, individuals exercising on a bike outdoors for 2 hr with ambient O_3_ levels > 0.08 ppm had greater lung function decrements as a group when they possessed a glutathione-S-transferase mu 1 (GSTM1) null genotype coupled with wild type NQO1 compared to other combinations of those 2 gene polymorphisms [10]. BMI has also been proportionally associated with O_3_-induced lung function decrements [11].

Lung function decrements are typically not induced by exposure to DE or PM alone in controlled study designs [12]. Controlled ambient PM exposure studies in humans have been used to examine biological responses as surrogates for understanding the health effects and mechanisms involved in the responses. A dose-dependent increase in total number of cells, neutrophils, and monocytes was observed in the lung lavage fluid in contrast to lung function. For instance, healthy human volunteers exposed to 300 μg/m^3^ DE for one hour with intermittent exercise resulted in marked systemic and pulmonary inflammatory responses [13],[14].

There is interest in examining whether ambient pollutant gases can modify the biological effect induced by PM [15]. A handful of controlled exposure studies have shown an interactive effect of PM and O_3_ exposure especially with blood and cardiac endpoints. For instance blood pressure increases have been noted with co-exposure of healthy and also asthmatic subjects to O_3_ and concentrated ambient particles (CAPS) [16]. Increased vasoconstriction in humans occurred upon exposure to O_3_ with CAPs [17]. Rodent models of multipollutant exposures have also been utilized to examine biological responses. In an allergic mouse model, both DE and O_3_ were needed to increase lung resistance, which was not observed with individual pollutant exposures [18]. Exposures of rats to resuspended DE particles and O_3_ induced changes in some aortic mRNA [19] suggesting changes induced by pollutant components traversing the lung.

There is the possibility that exposure to one pollutant may sensitize an individual in a manner that a biological response from an exposure to a second pollutant may be enhanced. Sequential O_3_ and DE exposures in healthy young adult participants in a controlled exposure setting has demonstrated that an initial exposure to 300 μg/m^3^ DE for 1 hr followed 5 hr later by a 2 hr exposure to 0.2 ppm O_3_ induced more lung inflammation (e.g. neutrophils) than DE alone with no O_3_ exposure [20]. The same research group, using a similar exposure regimen, showed that a prior exposure to DE increased O_3_-induced lung inflammation relative to O_3_ exposure alone [21]. However, these studies did not report on lung function changes. Combinations of exposure to sulfuric acid aerosols and O_3_ have demonstrated slightly additive lung function changes in asthmatic subjects [22] but not in healthy individuals [23]. This study primarily examined whether combinations of exposures to O_3_ and DE can induce additive or synergistic effects related to lung function changes in healthy, normal adults, whether exposure to DE alters the response to a subsequent exposure to O_3_ approximately 20 hrs later, and whether GSTM1 genotype status has an effect on the lung function responses.

## 2 Materials and Methods

### 2.1 Population characteristics

15 healthy, non-smoking volunteers (4 female, 11 male) completed all four Day 1 exposure regimens (a-d; described below) out of 25 subjects enrolled. Volunteers ranged in age from 23 to 36 (mean 27), had no history of asthma, chronic respiratory or acute illnesses within 4 weeks, and normal lung function (greater than 75% of predicted values for FVC, FEV1, and FEV1/FVC ratio). The volunteers refrained from taking non-steroidal anti-inflammatory medications 48 hr prior to each exposure. The use of vitamin C and E were not permitted for 2 weeks prior to exposure. [See Additional file 1 (Subject Selection and Participation Criteria) for full subject inclusion and exclusion criteria and subject participation requirements as well as the study purpose]. Participants were informed of the procedures and likely potential risks and each signed a statement of informed consent. Subject participation was strictly voluntary and they could withdraw at any time. The protocol and consent form were approved by the University of North Carolina School of Medicine Committee on the Protection of the Rights of Human Subjects and the US EPA [ClinicalTrials.gov # NCT01874834]. Once consented, volunteers were trained to correctly perform pulmonary function tests, BMI calculated, and participants exercised on a recumbent bicycle to determine a load that would elicit minute ventilation of 25 L/min/m^2^ body surface area (BSA) (~50 L/min for most subjects). BSA and BMI (in kg/m^2^) was calculated by the spirometry software from a subject’s height and weight. GSTM1 status was determined from isolated peripheral white blood cell DNA (QIAamp DNA Mini Kit (Qiagen Inc., Valencia, CA)) using RT PCR [24]; blood was drawn via standard venipuncture during the training day before any exposures occurred. One subject did not consent to genotyping and was not utilized in the analyses by genotype group. Subject characteristics are presented in Table 1.

**Table 1:** Subject characteristics of participants in the study

**Gender**	**Race**	**Age**	**BMI**	**GSTM1 status**
f	c	24	26.84	Pos
f	bl	27	32.16	Pos
m	c	37	24.19	Pos
m	c	27	24.37	Null
m	bl	36	28.47	Null
m	c	27	27.08	Pos
m	hisp	24	29.94	Pos
m	hisp	24	27.15	Null
m	c	23	25.22	Nos
m	bl	23	29.37	Pos
f	c	25	25.66	Null
f	bl	29	38.06	Pos
m	c	30	21.22	Pos
m	c	28	28.94	Null
m	c	25	25.72	nd
**11 m/4f**	**9c/4bl/2hisp**	**27 ± 4**	**27.63 ± 3.95**	**9+/5-**

nd = not determined.

### 2.2 Study design

**Figure 1:**
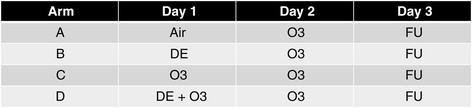
**Design of Study.** Subjects were exposed to target levels of either filtered air, 0.300 ppm O_3_, 300 ug/m^3^ DE, or combined DE (300 ug/m^3^) and O_3_ (0.300 ppm) on Day 1 for 2 hr. On Day 2 subjects were exposed to 0. 300 ppm O_3_ for 2 hr. During exposures on Days 1 and 2 subjects performed four 30 min cycles of rest/exercise at ~ 50 L/min. Day 3 was a follow-up (F/U) day for measurements and no pollutant exposures occurred.

This was a randomized crossover single blind study with 4 arms (designated as a-d) separated by at least 13 days between arms. Each arm consisted of 2 consecutive exposure days and a follow-up visit (Figure 1). On Day 1 participants were exposed for 2 hr with intermittent exercise to arm a) filtered air (FA) n = 3 (where n is the initial exposure); arm b) DE (300 ug/m3 target) n = 4; arm c) O_3_ (0.300 ppm target) n = 4; or arm d) DE + O_3_, n = 4. On Day 2 (~22 hr post Day 1 exposure) all research subjects were exposed to O_3_ (0.300 ppm target) for 2 hrs. A follow-up visit occurred on Day 3. No two subjects received the same sequence of arms (as there were 24 possible sequences). Prior to the first exposure (Day 1, pre), lung function was measured on a 10.2-L dry seal digital spirometer interfaced to a computer (SensorMedics Model 1022; SensorMedics; Palm Springs, CA) with subjects standing. Subjects then entered a stainless steel exposure chamber (approximately 1.83 m × 1.83 m × 2.44 m, ~8.17 m^3^; ~650 ft^3^/min flow; air change time ~ 27 sec), and were exposed to filtered air, DE, O_3_, or DE + O_3_ for 2 h. During the exposure, subjects exercised on a recumbent bicycle ergometer for 15 min intervals beginning after the first 15 min of exposure and resting for 15 min after each exercise time for a total of 1 hr of exercise per exposure. Ventilation rate was measured approximately 6 min into each exercise session and work load adjusted if needed to a target of 25 L/min/m^2^ BSA. Heart rate, ECG, and SpO_2_ (using pulse oximetry) were monitored continuously during the exposure. Immediately following the exposure (Day 1, post), subjects underwent pulmonary function testing. Lung function measurements were obtained once an hour for the next 4 hrs. On Day 2, the procedures described for Day 1 were repeated except that participants entered a different chamber (~2.39 m × 2.16 m × 3.20 m, ~ 16.52 m^3^; 450 ft^3^/min) for an O_3_ exposure. The following morning (Day 3, follow-up), there was a final round of pulmonary function testing. Subjects who had a large Day 1 decrement in lung function were not exposed on Day 2, but did return for follow-up lung function testing.

### 2.3 Exposure conditions

All exposures were carried out at the EPA Human Studies Facility in Chapel Hill, NC. The targeted exposure atmosphere was approximately 40 ± 10% RH and 22 ± 2°C. Outdoor air was passed through an air purification system consisting bed of Purifil (potassium permangate on alumina), a bed of Purikol (activated charcoal), dehumidified, and passed over an unheated bed of Hopcalite and then through a HEPA filter to ensure no detectable presence of pollutant gases (O_3_, NO, NO_2_, SO_2_, CO, and hydrocarbons with the exception of methane) in the FA. The air was then heated, humidified, and passed through a second HEPA filter before O_3_ and DE were added. The concentrations of the pollutant gases and PM were monitored continuously. The DE was generated from a Cummins diesel engine/generator QSB7 series (model DSGAA) running at 50 percent load that was located outside the Facility, and subsequently introduced into the exposure chamber after dilutions with humidified FA to give a chamber concentration target of approximately 300 μg/m^3^. The DE particle concentration was controlled by modulating the flow of medical grade compressed air into a Vortec Transvector Air Flow Amplifier (Model 901XSS; Cincinati OH) with real time TEOM monitoring of PM mass concentration. Diesel fuel used for the study was a commercially obtained ultra low sulfur (<15 ppm) fuel. Particle mass, numbers, and size characteristics were measured in real time by a DataRAM™, a Tapered Element Oscillating Microbalance (TEOM®), and a Condensation Particle Counter (CPC). The CPC TSI 3022A model (detection size limit 7 nm) was used for the first 11 subjects’ exposures, and a TSI 3775 model (detection size limit 4 nm) was used for the last 4 subjects’ exposures for detection of particle number concentration. A Scanning Mobility Particle Sizer (SMPS™) (Shorewood MN) as also used to measure particulate characteristics during subject exposures. The SMPS data were used to determine particle size distribution as a number median diameter in all size bins. The number median diameter is the particle size (μm) bin where the median of the total count number occurs. Particle size distribution was determined at regular intervals. A Versatile Air Pollution Sampler (VAPS) (URG, Chapel Hill NC) configured with one quartz and two teflon filters with flow controlled by a mass flow controller sampled inlet air to the chamber. After exposure and filter weight determination, filter samples were analyzed for chemical composition of particles. Elemental (EC) and Organic (OC) Carbon content were determined using a thermo-optical method based on sequential pyrolytic vaporization and detection by transmittance using a carbon analyzer (Model 107A, Sunset Laboratory Inc., Hillsborough, NC) [25]. A bacterial endotoxin characterization test on particles was conducted by Cape Cod Inc. (East Falmouth, Massachusetts) using a Limulus polyphemus gel clot method to test for endotoxin contamination following their standard operating procedures.

Ozone was generated by passing USP grade oxygen through a silent arc O_3_ generator (Innovatee Geratetechnmik GmbH, Rheinbach Germany) (Model CMG 10–5 for the DE chamber (Day 1) and Model 500 for the O_3_ chamber on Day 2). For Day 1 exposures O_3_ entered the filter air stream before DE. Concentrations were controlled by a computer feedback to the O_3_ generator with a 0-5 V signal allowing internal modulation control of the O_3_ generator. Both DE and O_3_ were delivered into the exposure chamber within 8 sec to minimize DE loss. Increased production of O_3_ was needed when DE was present as NO reacted with O_3_.

### 2.4 Statistics

The primary hypothesis of this study was to examine whether combinations of exposures to O_3_ and DE induce synergistic effects on lung function and whether exposures to DE sensitize individuals for a larger response to O_3_ than does the exposure by air. To evaluate changes between pollutant and filtered air exposures we used a full two-factor (arm b plus arm c (O_3_ plus DE), and arm d (O_3_ + DE)) mixed effects model with a subject-specific random intercept to account for repeated measures. To examine the first hypothesis with respect to synergism of effects, changes in lung function were examined following Day 1 exposures. To examine whether exposures to DE sensitize individuals for a larger response to O_3_, lung function was examined following O_3_ exposure on Day 2. In both analyses changes in lung function were contrasted to null hypothesis of no change relative to pre exposure baseline and relative to air exposure as control. Prior to the analysis, lung function endpoints FEV1 and FEV were normalized and expressed as percent change from the pre-exposure values (Post/Pre-1). All 15 subject subjects completed four exposures on Day 1. Following O_3_ exposure on day 1 some subjects responded with large drop in lung function and were not asked to undergo a Day 2 O_3_ exposure. On day 2, 15 subjects completed O_3_ exposures following air and DE (arms a and b, respectively), 14 of these completed O_3_ exposure following O_3_ on Day 1 (arm c), and 11 completed Day 2 O_3_ exposure following of the combined O_3_ & DE exposure (arm d). We used all available data in the analysis and did not account for possibly non-random missing values in subjects who responded strongly to O_3_ or O_3_ & DE on day 1. Estimated mean changes in lung function within each exposed group at each time point are expressed as mean change ± standard error, and a p-value where applicable. All statistical analyses were performed using R statistical software, with nlme and gmodes packages to estimate the effect (version 3.0.1; R Developement Core Team; http://www.r-project.org/). A *p* value of less than 0.05 was considered statistically significant.

## 3 Results

### 3.1 Exposure parameters

The subjects were exposed to 290 μg/m^3^ DE and 0.298 ppm O_3_ exposure on average during individual pollutant exposure days (Table 2). On co-exposure days, the values were very similar; 291 μg/m^3^ and 0.296 ppm for DE and O_3_ respectively. The values for other measured parameters (Table 2; CO, and TH) were also similar for the DE and DE + O_3_ exposures. When both O_3_ and DE were present together, there was a decrease in NO and a concomitant increase in NO_2_. On Day 2, when subjects were exposed to O_3_ alone, the concentration was maintained within 0.010 ppm of the targeted concentration. The median DE number and volume particle size (64 nm and 200 nm, respectively) did not change with O_3_ present (number size 66 nm and volume size 200 nm, respectively). There were 731 and 734 (x10^3^) particles/cc for DE and DE + O_3_ exposures, respectively when one CPC model was used (n = 11); the numbers increased when the second model was used (n = 4) due to a different detectable size limit. Particle EC and OC concentrations on sample filters were 39.2 ± 4.6 ug/cm^2^ and 14.2 ± 3.5 ug/cm^2^, respectively, i.e., ~ 3:1 ratio. PM from both DE and DE + O_3_ exposures had nondetectable levels of endotoxin.

**Table 2:** Mean Day 1 pollutant physicochemical parameters during the four exposure scenarios

**Parameter**	**Air**	**O**_**3**_	**DE**	**DE + O**_**3**_
**PM (μg/m**^**3**^**)**^**a**^	1.0 ± 1.5	1.0 ± 1.1	297. 1 ± 20.8	294.3 ± 20.1
**PM # (x10**^**3**^**/cc)**	<1	<1	731 ± 90^b^ (11)	734 ± 50^b^ (11)
			977 ± 55^c^ (4)	903 ± 306^c^ (4)
**Median PM size**				
**Volume (μm)**	-----	-----	0.200 ± 0.007	0.200 ± 0.007
**Number (μm)**^**d**^	-----	-----	0.064 ± 0.006	0.066 ± 0.003
**O**_**3**_**ppm**	0.00 ± 0.00	0.30 ± 0.00	0.01 ± 0.00	0.30 ± 0.00
**CO (ppm)**	0.09 ± 0.10	0.09 ± 0.10	2.58 ± 0.25	2.51 ± 0.32
**NO (ppm)**	0.00 ± 0.01	0.01 ± 0.03	1.58 ± 0.26	0.03 ± 0.04
**NO2 (ppm)**	0.00 ± 0.00	0.00 ± 0.00	0.16 ± 0.05	1.72 ± 0.22
**THC (ppm)**	2.05 ± 0.10	1.98 ± 0.10	2.35 ± 0.14	2.24 ± 0.19
**SO2 (ppm)**	<0.010	<0.010	0.013	<0.010

mean ± Std Dev except for Median PM size; n = 15, except for PM# where n is indicated.

^a^Gravimetric weight; average of two teflon filters.

^b^CPC Model TSI 3022A.

^c^CPC Model TSI 3775.

^d^particle size (μm) bin where the median of the particle counts occurred.

### 3.2 Lung function after exposures to pollutants on Day 1

**Figure 2:**
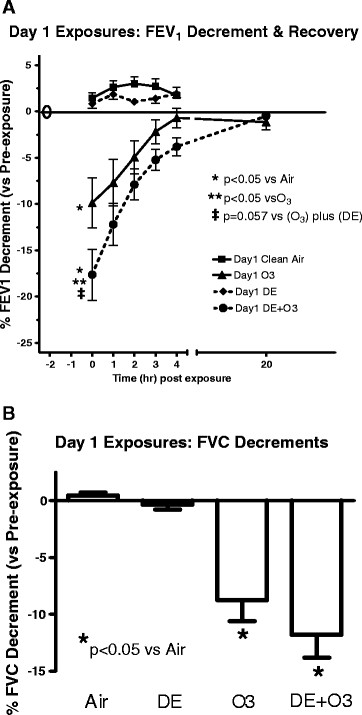
**Changes in FEV1 (A) and FVC (B) after Day 1 pollutant exposures.** Subjects performed pre-exposure spirometry immediately before a 2 hr exposure (filtered air, O_3_, DE, or DE + O_3_). Spirometry was performed immediately post-exposure and every hour post-exposure for 4 hr. FVC Data in (B) derived from the immediately post time point only. Data in the figure are presented as the mean ± SEM of each group at that time point. *p < 0.05 vs air exposed; **p < 0.05 vs O_3_ exposure alone; ‡ p = 0.057 vs the sum of the O_3_ plus DE exposure FEV1 changes using Mixed Effects Model testing as described in the materials and methods section.

Fifteen subjects completed all four of the Day 1 pollution exposure scenarios. In these subjects, decreases (relative to their pre-exposures values) in FEV1 were not observed immediately after exposure to either FA or whole DE (Figure 2A). Measurement of lung function revealed no decrement in DE or air exposed subjects for up to 4 hrs post-exposure. Exposure to O_3_ induced an average decrement of 9.9 ± 2.5% measured immediately post-exposure (p < 0.05 vs air exposure). Exposure to the combination of O_3_ and DE induced a decrement of 17.6 ± 4.6% and was statistically different from both air and O_3_ alone exposure (p < 0.01). A greater than additive effect of the DE and O_3_ exposure (arm d) on FEV1 was examined by statistical comparison to the FEV1 changes following O_3_ (arm c) added to the change following DE exposure (arm b). The analysis had a p value of 0.057, close to statistical significant. FEV1 values returned to pre-exposure values by 4 hr post-exposure in the O_3_ and DE + O_3_ groups at similar rates.

Among the Day 1 exposures statistically significant (p < 0.01) FVC decrements were observed in O_3_-exposed subjects (8.7 ± 2.3%) and DE + O_3_ exposed subjects (11.8 ± 3.0%) immediately post-exposure compared to FA exposures (Figure 2B). The FVC decrements immediately post O_3_ compared to post DE + O_3_ exposure were not statistically different (p = 0.10), nor was greater than additive effects observed when FVC changes in arm d were compared to the sum of FVC changes post O_3_ and DE. Exposure to either air or DE alone did not alter FVC from pre-exposure values. FVC values returned to pre-exposure levels by 4 hrs post-exposure for all exposure groups with no difference in the recovery rate (data not shown).

**Figure 3:**
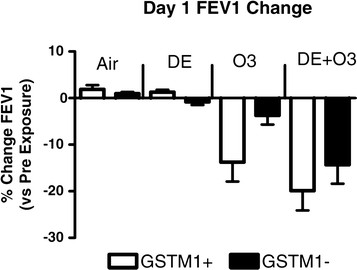
**Day 1 Changes in FEV1 immediately after a Day1 exposure in GSTM1- individuals.** Mixed Effects Model testing was used for statistical analysis.

Analysis stratified by GSTM1 genotype showed relatively small and non-significant decrease in FEV1 in GSTM1null individuals (n = 5) (0.8 ± 0.7%) immediately post DE exposure compared to individuals with a GSTM1+ genotype who did not have a decrease (FEV1 increased 1.3 ± 0.5%; n = 9) (Figure 3). Immediately after the O_3_ exposure, the group of GSTM1null genotype individuals had a smaller, and statistically non-significant, decrease in FEV1, 3.70 ± 2.0% compared to a 13.7 ± 4.2% decrement in the GSTM1+ group. Using linear regression, an individual’s BMI did not significantly correlate with their observed O_3_-induced or DE and O_3_-induced FEV1 or FVC change.

### 3.3 Lung function after exposure to O_3_ on Day 2

**Figure 4:**
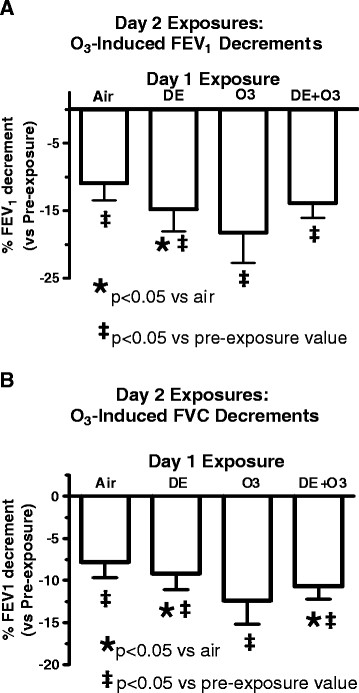
**Changes in FEV1 (A) and FVC (B) immediately post Day 2 O**_**3**_**exposure (0. 300 ppm).** *p < 0.05 vs air exposed values using Mixed Effects Model testing.

**Figure 5:**
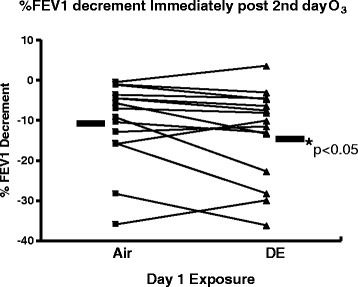
**Individual changes (n = 15) in FEV1 immediately after Day 2 O**_**3**_**exposure for individuals exposed to either air or DE on day 1.** *p < 0.05 vs air exposure using Mixed Effects Model testing.

After Day 1 exposures, subjects returned to the facility and most were exposed to about 0.300 ppm O_3_. For lung function measured immediately post-exposure, subjects in arm a (exposed to air on day 1) had a statistically significant O_3_-induced decrease in FEV1 (10.9 ± 2.6%) and FVC (7.8 ± 1.8%) immediately post-exposure when compared to pre-exposure values (Figure 4A and 4B). Exposure to O_3_ after the previous day exposure to DE (arm b) induced statistically significant decrements of 14.7 ± 3.3% for FEV1 and 9.2 ± 1.9% for FVC (both p < 0.05). The Day 2 O_3_-induced FEV1 and FVC decrements for each subject after the Day 1 exposure to DE compared to air showed that prior exposure to DE on Day 1 induced a statistically significant greater FEV1 decrement on Day 2 (p < 0.05) (Figure 5).

Some subjects were not sequentially exposed to Day 2 O_3_ either due to their request or due to their large decrement in FEV1 immediately after either O_3_ (n = 1) or DE + O_3_ (n = 4) on Day 1 because a second sequential O_3_ exposure within 24 hr is expected to typically induce an even larger FEV1 decrement than the first day exposure in most subjects. Consequently, for those five subjects Day 2 procedures were follow-up measurements and observations, similar to Day 3 measurements for the other 10 subjects. Subjects in this study had a greater O_3_-induced lung function decrements if they received two consecutive days of O_3_ exposure (arm c); consecutive O_3_ exposures induced a statistically significant greater decrement in FEV1 (18.2 ± 4.5%) than the decrement associated immediately after the first day exposure, (i.e., 10.3 ± 3.3%,) (p < 0.05) (Figure 4A) or compared to the Day 1 air exposure, then O_3_ on Day 2 (i.e., 10.9 ± 2.6%) . The FVC decrement (7.8 ± 1.8%) on Day 2 following air (arm a) (Figure 4B) was similar to the Day 1 FVC decrement (8.7%) after O_3_ (Figure 2B). For subjects exposed to DE + O_3_ (arm d) the FEV1 decrement immediately after exposure to O_3_ on Day 2 was 13.8 ± 2.2% , which was similar to the decrement in these subjects the previous day immediately after exposure (i.e., 17.6 ± 4.6%). The FVC decrement on Day 2 (Figure 4B) was similar to the Day 1 FVC decrement as well (Figure 2B). Both FEV1 and FVC values returned to pre-exposure values by 4 hr post exposure (data not shown).

When FEV1 responses to the Day 2 O_3_ exposure were segregated by GSTM1 genotype, it was observed that those subjects with the null genotype had a smaller decrement (3.8 ± 3.8%) than positive genotypes (14.9 ± 3.6%) after the FA exposure day; however this difference was not significant. This is a consistent effect with the observed Day 1 responses to O_3_ exposure between the two groups. No other Day 2 FEV1 differences were observed between genotypes for the other 3 exposure regimens from Day 1.

## 4 Discussion

This study examined alterations in lung function after co-exposures of DE and O_3_, as well as sequential exposures to DE and O_3_ separated by one day. For the former exposure regimen, DE alone did not induce lung function as measured by FEV1 and FVC decrements, but O_3_ did induce decrements after 2 hr reaching statistical significance for FEV1. Exposure of the subjects to the combination of the two pollutants (arm d) induced a greater FEV1 decrement than either pollutant alone. If the pollutant-induced FEV1 changes are added together, i.e., the sum of the changes in FEV1 post O3 and post DE exposures, and compared to the combined exposure (arm d), the p value of 0.057 is obtained and is close to statistical significance (Figure 2A). A possible explanation for the synergistic effect induced by DE + O_3_ co-exposure may be that the lung function change is related to the chemical composition in the atmosphere containing O_3_ and DE being different from DE or O_3_ alone. DE-derived particle size did not change with the presence of O_3_, and chamber temperature and humidity were kept similar between the two exposure regimens, suggesting that particle deposition pattern in the lung was not likely altered due to a size change. However, the possibility that other PM physicochemically related characteristics were altered, such as a change in charge or hydrophobicity that could alter the deposition pattern cannot be excluded. For all exposures regimens, lung function returned to pre-exposure values by 4 hrs after the end of the exposure, similar to the well characterized and transient nature of O_3_-induced lung function changes.

With respect to component changes in the gas phase relating to the greater than additive effect of O_3_ and DE on FEV1 decrement, the NO_2_ level increased while the NO level decreased. However, NO_2_ alone generally does not induce lung function changes even up to 4 ppm in healthy individuals [26] though we cannot totally exclude the possibility that it contributed to changes in lung function seen during co-exposure to DE and O_3_. O_3_ concentrations were kept at ~ 0.300 ppm with or without DE present, so the only other components with potentially altered concentrations were derived from DE. If lung function responses to O_3_ exposure are mediated at least in part via C-fiber activation [27] then chemical components affecting C-fibers activity directly (but below a threshold for discharge), or indirectly by, e.g., lowering the fiber threshold for electrical discharge, might be expected to induce the synergistic change in FEV1 seen upon concurrent O_3_ and DE exposure. Some carbonyls, (e.g., acrolein) and carboxylic acids (e.g., acetic acid) have been implicated in affecting pulmonary C-fiber activity [28]–[30]. There are several aldehydes identified in DE including acrolein, and there is increased formation of carbonyls and carboxylic acids observed upon O_3_ reacting with DE particles surface components [31]. There is evidence for a possible increased carbonyls and/or carboxylic acids formation and induction of C-fiber sensitization in the DE and O_3_ atmosphere, but other classes of compounds cannot be eliminate either.

Subjects in this study receiving DE exposure the day before the Day 2 O_3_ exposure had greater FEV1 decrements than when they had a Day 1 clean air exposure (Figure 5). It is uncertain whether only the DE gas phase or particle phase, or both phases, are needed to induced the augmented response to a subsequent O_3_ exposure. Most previous studies examining sequential exposure effects of air pollutants on O_3_-induced lung effects changes were typically performed within 6 hr of the initial exposure [20],[21],[32]. A report demonstrated that prior exposure for 2 hr to 0.6 ppm NO_2_ with intermittent exercise (~40 L/min) did not significantly alter lung function in the young, healthy subjects; however compared to air as the initial exposure, the pre-exposure to NO_2_ induced a 1.8% greater FEV1 decrement upon a 2 hr exposure to 0.300 ppm O_3_ 3 hr post NO_2_ exposure [32]. Another study reported that a same day follow up to approximately 0.200 ppm O_3_ added to the lung inflammatory response (i.e., increased neutrophilia and myeloperoxidase) induced by an initial 300 ug/m^3^ DE exposure compared to an initial air exposure; but lung function was not examined in that study [21]. A possible mechanism involved with the augmented FEV1 change to O_3_ on Day 2 in this study may involve, at least in part, altered production of prostaglandins E_2_ (PGE_2_) and F_2α_ (PGF_2α_) which have been associated with O_3_-induced lung function decrements [33]. Both prostaglandins can alter lung C-fiber activity directly inducing discharge and/or altering the threshold for discharge to an agonist [34] The possible altered AA metabolism in response to sequential DE and O_3_ exposures (i.e., increased AA release, decreased re-uptake, and COX-2 protein for increased conversion of AA to PGE_2_ and F_2α_), based upon *in vivo* and *in vitro* findings [35]–[39], may explain, at least in part, the observed greater FEV1 decrement. It should be noted that an adequate duration of time is needed for COX-2 protein to be upregulated [40]. Additionally the literature suggests that the DE particle phase is involved in this possible biochemical mechanism (i.e., increased COX-2), but does not necessarily negate the importance of DE gas phase components.

Factors that affect an individual’s sensitivity to O_3_-induced lung function decrements are not clearly understood. Increased BMI has been linked to a greater O_3_-induced lung function decrement though predominantly driven by females with BMI > 25 [11]. In our study no statistically significant changes were observed in the whole population, and with only four female study participants the analyses was likely underpowered. Certain genotypes, including GST isozymes, have been examined as possible biomarkers of sensitivity. Specifically for this study, GSTM1 genotype status was examined as the null genotype was not associated with a greater FEV1 decrease upon exposure of young, normal, healthy adults to a relatively low 0.06 ppm O_3_ concentration for 6.6 hr with exercise (24), though lung neutrophilia was associated with the allele deletion. In our study, the GSTM1- subjects had an approximately 10% smaller decrement to 0.300 ppm O_3_ exposure than GSTM1+ subjects in arm c Day 1 and arm a Day 2, though the difference did not reach statistical significance. However, no increased resistance to development of an FEV1 decrease was observed in the GSTM1- group upon the co-exposure to DE and O_3_, possibly due to a similar sensitivity to DE components or DE components that were influenced by O_3_.

The modulation of O_3_-induced lung function changes by DE exposure raise the possibility that such interactions may occur from exposures to ambient levels of these pollutants. Additionally the components in DE that altered the magnitude of the O_3_-induced FEV1 changes may be also present in our environment and potentially derived from natural or anthropomorphic sources and not exclusively from DE. It is unclear at what ambient concentrations DE may affect responsiveness to O_3_ exposure, and also the threshold of O_3_ level that induces lung function decrements; recent literature suggest it may be as low as the 0.06-0.08 ppm range [24],[41]. Additionally populations sensitive to the DE-induced lung function effects have not been fully identified, and remains an ongoing issue.

## Competing interests

The authors declare that they have no competing interests.

## Authors’ contributions

MCM was a principal investigator for part of the study duration, contributed substantially to the study design and protocols, prepared the initial draft of this manuscript, assisted with subject supervision, analyzed data, and performed internal clearances. TS was also a principal investigator for part of the study duration and protocols, assisted with the writing of this manuscript and data analyses, assisted with subject supervision; MC contributed substantially to the study design and protocols, assisted with subject supervision, and analyzed data; MS was the study coordinator, contributed substantially to the protocol, and performed genotyping; MB and TSM had the primary subject supervision and monitoring responsibilities and contributed to the study protocol; JB contributed substantially to the study design and protocol, contributed substantially to the initial draft of this manuscript, and was responsible for the pollutant exposure engineering oversight and quality assurance; DDS and RBD contributed substantially to the study design and protocols, contributed substantially to the review of drafts of this manuscript. All authors read and approved the final manuscript.

## Additional file

## Supplementary Material

Additional file 1: Subject selection and participation criteria. Click here for file
