# Supplementary material for: Diesel exhaust modulates ozone-induced lung function decrements in healthy human volunteers
Source: Part Fibre Toxicol. 2014 Sep 2;11:37. doi: 10.1186/s12989-014-0037-5 (PMC4354281; doi:10.1186/s12989-014-0037-5)
Supplement: Additional file 1: — Subject selection and participation criteria. [file s12989-014-0037-5-S1.pdf]

Supplement to:

Diesel Exhaust Modulates Ozone-induced Lung Function Decrements in Healthy Human Volunteers

AUTHOR NAMES: Michael C. Madden[1], Tina Stevens*[1], Martin Case[1], Michael Schmitt[1], David Diaz-Sanchez[1], Maryann Bassett[1], Tracey Montilla[1], Jon Berntsen[2], Robert B. Devlin[1]

[1]EPHD, NHEERL, US EPA, Research Triangle Park, NC, USA 27711

[2]TRC Environmental, Chapel Hill, NC 27514

*Currently ORISE, Research Triangle Park, NC, USA 27711

AUTHOR ADDRESS: U.S EPA Human Studies Facility 104 Mason Farm Road Chapel Hill, NC 27599-7315

AUTHOR EMAIL ADDRESS: madden.michael@epa.gov; 919-966-6257

Running Title: Diesel exhaust and ozone effects on lung function

Disclaimer: This report has been reviewed by the National Health and Environmental Effects Research Laboratory, United States Environmental Protection Agency and approved for publication. Approval does not signify that the contents necessarily reflect the views and policies of the Agency nor does mention of trade names or commercial products constitute endorsement or recommendation for use. None of the authors has any actual or potential competing financial interests.

Materials and Methods

Subject Selection and Participation Criteria:

Inclusion Criteria:

1. Healthy men and women between 18 and 55 years of age
2. Physical conditioning allowing intermittent, moderate exercise for 2 hours
3. Normal lung function:
   a. FVC > 75 % of that predicted for gender, ethnicity, age and height.
   b. FEV_1 > 75 % of that predicted for gender, ethnicity, age and height.
   c. FEV_1/FVC ratio > 75 % of predicted values.
4. Oxygen saturation > 96 %.

Exclusion Criteria:

1. A history of acute and chronic cardiovascular disease, chronic respiratory disease, diabetes, rheumatologic diseases, immunodeficiency state, and acute respiratory illness within 4 weeks.
2. Subjects who are asthmatic or have a history of asthma.
3. Allergic to chemical vapors or gases.
4. Any allergic symptoms during the time of participation in the study
5. Female subjects who are currently pregnant, attempting to become pregnant, or breastfeeding.
6. Subjects unwilling or unable to stop taking vitamin C or E or medications which may impact the results of the ozone challenge (such as, systemic steroids and beta blockers) at least 2 weeks prior to the study and for the duration of the study. Medications not specifically mentioned here may be reviewed by the investigators prior to a subject's inclusion in the study.
7. Current and past smokers within 1 year.
8. Uncontrolled hypertension (> 150 systolic, > 90 diastolic).
9. Subjects who do not understand or speak English
10. Subjects unable to perform the moderately active exercise required for the study.
11. Subjects with a history of skin allergies to adhesives used in securing heart rate monitor electrodes.
12. Unspecified diseases or conditions, which in the judgment of the investigator might influence the responses to the exposures, will be a basis for exclusion.
13. Subjects unwilling to stop taking over-the-counter pain medications such as aspirin, Advil, Aleve or other non-steroidal anti-inflammatory medications ("NSAIDS") for 48 hr prior to the exposures and post-exposure visits.
14. Subjects with a marked baseline prolongation of QT/QTc interval (e.g., repeated demonstration of a QTc interval >450 milliseconds (ms))

In order to participate in this study, subjects were asked to:

- Avoid smoke and fumes for 24 hours before all visits.
- Avoid drinking alcohol 24 hours before all visits.
- Avoid strenuous exercise for 24 hours prior to and after all visits.
- Avoid the use of ozone-based home air purifiers during study participation.
- On the exposure days, eat a light breakfast.
- Refrain from all over the counter anti-inflammatory agents including those for allergies for a period of 48 hrs prior to exposure.

Purpose of the Study (quoted from the UNC-Chapel hill and US EPA approved study protocol): "The purpose of this study is, first, to examine whether DE can alter lung and cardiovascular responses to O3 exposure when given a day before or during O3 exposure; second, to investigate if co-exposure of DE and O3 on day 1 augments the lung function decrements following a subsequent O3 exposure (day 2); third, to investigate if two consecutive days of O3 affect individual cardiovascular responses such as changes in heart rate variability (HRV) and blood pressure (BP), to ozone in young healthy adults. We will measure spirometric lung function in association with 2-hour exposures to O3. Results will be analyzed for effects of each or combinations of the above on changes in lung function."
